# Association of lung function with functional limitation in older adults: A cross-sectional study

**DOI:** 10.1371/journal.pone.0253606

**Published:** 2021-06-29

**Authors:** Yu Gao, Liang Shen, Runqing Zhan, Xiaoxu Wang, Huanhuan Chen, Xiaoli Shen

**Affiliations:** 1 Department of Epidemiology and Health Statistics, The School of Public Health, Qingdao University, Qingdao, Shandong, China; 2 Department of Business School, Qingdao University of Technology, Qingdao, China; 3 Qingdao University Affiliated Hiser Hospital, Qingdao, China; Universidad Miguel Hernandez de Elche, SPAIN

## Abstract

**Introduction:**

Impaired lung function is independently associated with higher rates of disability, however, few studies have examined the association between lung function and functional limitation. This study aimed to assess this association and dose-response relationship in older adults.

**Methods:**

Data from the National Health and Nutrition Examination Survey (2007–2012) was used as a cross-sectional study. Lung function was determined by Forced expiratory volume in 1 second (FEV_1_) and forced vital capacity (FVC). Functional limitation in older adults was identified by six self-reported questions on physical function. 3070 adults aged 60 and over were enrolled in our study. Logistic regression models and restricted cubic spline models were applied to examine the association between lung function and the risk of functional limitation.

**Results:**

FEV_1_ and FVC were inversely associated with the risk of functional limitation. In the full adjusted model, compared with the lowest tertile of FEV_1_, the odds ratios (95% confidence intervals) of functional limitation for tertile 2 and tertile 3 were 0.5422 (0.3848–0.7639) and 0.4403 (0.2685–0.7220), and the odds ratios (95% confidence intervals) of functional limitation for tertile 2 and tertile 3 of FVC were 0.5243 (0.3503–0.7848) and 0.3726 (0.2072–0.6698). Furthermore, an inverse association persisted after stratified analysis by gender and sensitivity analysis. Dose-response analyses showed that the odds of functional limitation declined with increase in FEV_1_ and FVC in a nonlinear manner.

**Conclusions:**

Lung function was inversely associated with functional limitation among older adults.

## Introduction

Functional limitation, that is restriction in basic mobility, often occurs among the geriatric population during progressive aging [1]. Due to physiological changes and underlying chronic diseases, physical function on mobility gradually degenerates over time and results in functional limitation, even disability [2–4]. Functional limitation is related to numerous risk factors, including alcohol intake, pain, obesity, physical activity, muscle strength, sensory impairments, and accident [5–8]. Maintaining physical function to boost activity is critical for living independently, maintaining the quality of life, and avoiding disability. Thus, exploration of the protective factors of functional limitation in older adults is important and valuable for public health.

Prior studies have established that lung function declined progressively with aging [9, 10], while good lung function was thought to benefit a healthier and longer life [11]. Low birthweight, ambient air pollution, obesity, and asthma were confirmed as determinants of lung function impairment among never-smokers [12]. Lung function impairment may lead to reduced muscle mass and altered metabolism [13, 14], which further progressed to muscle atrophy and muscle dysfunction [15], finally limiting exercise performance [16].

Several epidemiological studies have demonstrated that chronic obstructive pulmonary disease (COPD), a major cause of lung function impairment, was positively associated with disability [17–21]. It is generally accepted that progression from aging to functional disability in the elderly may be a slow process, while functional limitation foreshadow functional disability in future [1]. Hence, we speculate that there exists a negative association between lung function and functional limitation. This association has been observed in previous studies in the patients with lung functional impairment [22, 23]. However, no research has focused on the association between lung function and function limitation in older adults. In order to judge the true impact of lung function on functional limitation, we conducted a cross-sectional study, using a representative general population, to evaluate the association and dose-response relationship of lung function with functional limitation in the elderly.

## Materials and methods

### Study population

The National Health and Nutrition Examination Survey (NHANES) collects data by two-year cycles on health and nutrition with a stratified multistage probabilistic sample design. The data are from a nationally representative sample of US adults. The Center for Disease Control’s National Center for Health Statistics conducts surveys with in-person interviews and physical examinations in a mobile examination center (MEC). The current study was approved by the National Center for Health Statistics Research Ethics Review Board and informed consent was obtained from all participants. Data from three cycles of NHANES (2007–2008, 2009–2010, 2011–2012), with a total sample of 30244 people, were analyzed in this study. We excluded individuals aged younger than 60 years (n = 24226) and those lacking lung function data (n = 2431), those missing height (n = 19) or functional limitation information (n = 498). Finally, 3070 participants were included in this study.

### Lung function

An Ohio 822/827 dry-rolling seal volume spirometer was used to record the exhaled volume and flow rate. Eligible participants were asked to take a deep breath and then exhale with maximum effort, called the forced expiratory maneuver. Spirometry procedures conformed to the recommendations of the American Thoracic Society (ATS) [24]. Expiration was required to least 6 seconds. Forced expiratory volume in 1 second (FEV_1_) and forced vital capacity (FVC) were analyzed in this study. As standing height was the most important factor affecting lung function, FEV_1_ and FVC were adjusted for height squared to obtain more precise and stable measures compared to original measurements alone, based on recommendations from previous studies [25–27].

### Functional limitation

The definition of functional limitation was based on previous studies [28, 29]. All participants were asked to answer questions regarding the extent of difficulty of functional activities, with options including “No difficulty”, “Some difficulty”, “Much difficulty”, “Unable to do” or “Do not do this activity”. The choice “Do not do this activity” was deemed to represent missing data. Self-report of “much difficulty” or “unable to do” one or more of 6 tasks listed below, was considered as functional limitations. Tasks included “walking for quarter of a mile”, “walking up 10 steps”, “stooping/crouching/kneeling”, “standing straight up from an chair”, “lifting or carrying 10 pounds in weight”, and “walking to another room on the same floor”.

### Covariates

The covariates examined in this study included age, gender, race, education level, marital status, body mass index (BMI), smoking status, alcohol drinking status, household income, work activity, recreation activity, and medical history of hypertension, diabetes mellitus, stroke, arthritis, gout, and cancer. Race was specified as Mexican American, other Hispanic, Non-Hispanic White, Non-Hispanic Black, and other race. Education level was separated into “below high school”, “high school”, and “above high school”. Marital status was allocated in 3 categories: married/living with partner, widowed/divorced/separated, and never married. BMI grouping include: <25 kg/m^2^, 25 to<30 kg/m^2^, ≥30 kg/m^2^. Participants were questioned regarding smoking status and alcohol drinking status with questions of “have you smoked smoking at least 100 cigarettes in your life or not” and “do you have at least 12 alcoholic drinks/year or not”. Household annual income was divided as, less than $20,000 or not less than $20,000. Both work activity and recreation activity were classified as vigorous activity, moderate activity, or other. Hypertension, diabetes mellitus, stroke, arthritis, gout, and cancer were defined based on self-reported history of diagnosis.

### Statistical analyses

Given that three two-year cycles of NHANES (2007–2008, 2009–2010, 2011–2012) were combined, new weighting was constructed using one-third of the two-year MEC examination weights in the light of the analytical guideline of NHANES. We tested the normality of continuous variables with the Kolmogorov–Smirnov normality test. Mean ± SD for normally distributed continuous variables, median (interquartile range) for non-normally distributed continuous variables and number (percentage) for categorical variables were used to describe the respective characteristics in this study. Student’s t-tests or nonparametric test for continuous variables and Chi-squared tests for categorical variables were used to compare the two groups “with functional limitation” and “without functional limitation”. FEV_1_ and FVC were evenly divided into three levels according to tertiles, and the lowest tertile was set as reference. Binary logistic regression was conducted to estimate the association between lung function in different levels and functional limitation. Odds ratios (OR) and its 95% confidence intervals (CI) were given. Model 1 adjusted for age, gender, BMI, smoking status, and stroke, and model 2 additionally adjusted for race, education level, marital status, alcohol drinking status, household income, work activity, recreation activity, hypertension, diabetes mellitus, arthritis, gout, and cancer. We also performed analysis of linear trends by inputting median values of each tertiles into models as continuous variables. Restricted cubic spline regression was conducted to assess relationships between FEV_1_, FVC and functional limitation with knots of 5th, 50th, and 95th percentiles of the exposure distribution in model 2. The P value for non-linearity was determined by assessing whether the coefficients of the second splines were equal to 0 or not. In addition, we carried out stratified analysis on gender. All data analyses were performed using Stata version 15 (Stata Corp LP, College Station, TX). P value <0.05 on two-sided testing was considered to be statistically significant.

## Results

Characteristics of participants with functional limitation and without functional limitation were presented in Table 1. Individuals with functional limitation and without functional limitation comprised 13.5% and 86.5% of the total in older adults. Compared with participants without functional limitation, the means of FEV_1_ and FVC were both lower in the elderly with functional limitation: at 0.778 L/m^2^ and 1.060 L/m^2^, respectively. Participants with functional limitation tended to be older, to have a lower level of lower education level and income, and were more likely to be widowed/divorced/separated, more likely to suffer from obesity, loss of work and recreation activity, more likely to be smokers. Besides, those with functional limitation were more likely to have a history of hypertension, diabetes mellitus, stroke, arthritis, gout, or cancer.

**Table 1:** Characteristics associated with functional limitation among the elderly, NHANES 2007–2012.

Characteristics	With functional limitation	Without functional limitation	P value
**N, (%)**	413 (13.5)	2657 (86.5)	
**Age(year), median (interquartile range)**^**c**^	68 (10)	67 (10)	0.001
**Gender (%)**^**b**^			<0.001
Male	150 (36.32)	1413 (53.18)	
Female	263 (63.68)	1244 (46.82)	
**Race (%)** ^**b**^			0.124
Mexican American	58 (14.04)	305 (11.48)	
Other Hispanic	55 (13.32)	273 (10.27)	
Non-Hispanic White	192 (46.49)	1302 (49.00)	
Non-Hispanic Black	88 (21.31)	609 (22.92)	
Other race	20 (4.84)	168 (6.32)	
**Education (%)** ^**b**^			<0.001
Below high school	191 (46.36)	709 (26.70)	
High school	97 (23.54)	630 (23.73)	
Above high school	124 (30.10)	1316 (49.57)	
**Marital status (%)**^**b**^			<0.001
Married/Living with partner	218 (52.91)	1752 (65.99)	
Widowed/Divorced/Separated	177 (42.96)	765 (28.81)	
Never married	17 (4.13)	138 (5.20)	
**Household income (%)** ^**b**^			<0.001
<$20,000	152 (38.68)	483 (18.94)	
≥$20,000	241 (61.32)	2067 (81.06)	
**Body mass index (%)** ^**b**^			<0.001
<25 kg/m^2^	63 (15.29)	682 (24.69)	
25 to <30 kg/m^2^	135 (32.77)	1018 (38.34)	
≥30 kg/m^2^	214 (51.94)	955 (35.97)	
**Work activity (%)** ^**b**^			0.001
Vigorous activity	35 (8.47)	386 (14.53)	
Moderate activity	85 (20.58)	611 (23.00)	
Other	293 (70.94)	1660 (62.48)	
**Recreational activity (%)** ^**b**^			<0.001
Vigorous activity	7 (1.69)	299 (11.25)	
Moderate activity	85 (20.58)	957 (36.02)	
Other	321 (77.72)	1401 (52.73)	
**Smoking at least 100 cigarettes in life (%)** ^**b**^			<0.001
Yes	211 (51.21)	1338 (50.40)	
No	201 (48.79)	1317 (49.60)	
**Have at least 12 alcoholic drinks/year (%)** ^**b**^			<0.001
Yes	229 (57.83)	1776 (69.70)	
No	167 (42.17)	772 (30.30)	
**Hypertension (%)**^**b**^			<0.001
Yes	287 (69.49)	1445 (54.43)	
No	126 (30.51)	1210 (45.57)	
**Diabetes mellitus (%)** ^**b**^			<0.001
Yes	128 (32.49)	473 (18.28)	
No	266 (67.51)	2114 (81.72)	
**Stroke (%)** ^**b**^			0.001
Yes	27 (6.59)	85 (3.20)	
No	383 (93.41)	2569 (96.80)	
**Gout (%)** ^**b**^			0.003
Yes	45 (10.90)	179 (6.74)	
No	368 (89.10)	2476 (93.26)	
**Arthritis (%)** ^**b**^			<0.001
Yes	285 (69.01)	1055 (39.77)	
No	128 (30.99)	1598 (60.23)	
**Cancer or malignancy (%)** ^**b**^			0.937
Yes	71 (17.23)	453 (17.08)	
No	341 (82.77)	2200 (82.92)	
**FEV**_**1**_**(L/m**^**2**^**), mean (SD)**^**a**^	0.7783 (0.1959)	0.8756 (0.1994)	<0.001
**FVC(L/m**^**2**^**), mean (SD)**^**a**^	1.0604 (0.2424)	1.1914 (0.2557)	<0.001

Data was present in frequency (percentage), mean (SD), and median (interquartile range). FEV_1_ represents FEV_1_/height^2^, and FVC represents FVC/height^2^.

a quantitative variable tested by Student’s t-test.

b qualitative variable tested by Chi-square test.

c quantitative variable tested by Mann–Whitney U test.

Table 2 showed the association of lung function with functional limitation among the older participants according to tertiles of FEV_1_ and FVC. Under all the conditions considered: in the unadjusted model, in model 1 (adjusted for age, gender, BMI, smoking status, and stroke), or in model 2 (adjusted for all covariates), the results of logistic regression analyses were clearly statistically significant. Higher values of FEV_1_ and FVC were associated with decreased odds of functional limitation in the different models, compared with measurements in the lowest tertile. In adjusted multivariate analysis, the odds ratios (95% CIs) of functional limitation for tertile 2 and tertile 3 of FEV_1_ were 0.5422 (0.3848–0.7639) and 0.4403 (0.2685–0.7220), and were 0.5243 (0.3503–0.7848) and 0.3726 (0.2072–0.6698) for FVC, compared with the lowest tertile.

**Table 2:** Weighted ORs and 95% CIs for functional limitation according to tertiles of lung function, NHANES 2007–2012.

	Case/Participants	Crude	Model 1	Model 2
**FEV**_**1**_				
<0.76	192/928	Ref.	Ref.	Ref.
0.76 to 0.93	131/1048	0.4706 (0.3364–0.6583)**	0.5351 (0.3811–0.7512)**	0.5422 (0.3848–0.7639)**
>0.93	90/1094	0.2546 (0.1879–0.3450)**	0.4041 (0.2730–0.5980)**	0.4403 (0.2685–0.7220)**
P-trend		<0.001	<0.001	<0.001
**FVC**				
<1.04	202/933	Ref.	Ref.	Ref.
1.04 to 1.26	130/1036	0.4346 (0.3062–0.6167)**	0.5317 (0.3686–0.7671)**	0.5243 (0.3503–0.7848)**
>1.26	81/1101	0.1985 (0.1408–0.2799) **	0.3130 (0.2008–0.4878)**	0.3726 (0.2072–0.6698)**
P-trend		<0.001	<0.001	0.001

* P<0.05

**P<0.01. FEV_1_ represents FEV_1_/height^2^, and FVC represents FVC/height^2^.

Model 1 adjusted for age, gender, BMI, smoking status, and stroke.

Model 2 adjusted for more covariates: age, gender, race, education level, marital status, BMI, smoking status, alcohol drinking status, household income, work activity, recreation activity, hypertension, diabetes mellitus, stroke, arthritis, gout, and cancer.

The results of being functional limitation associated with lung function stratified by gender are displayed in Table 3. In male, the inverse association between FEV_1_ and functional limitation was significant in highest tertile across the univariate model, model 1, and model 2. Meanwhile, the inverse association between FVC and functional limitation was observed in the highest tertile. In female, the association of FEV_1_ and FVC with functional limitation remained inverse in unadjusted model and model 1. In model 2, the OR (95% CI) of functional limitation in highest tertile of FEV_1_ was 0.5432 (0.3149–0.9371), whereas the OR of functional limitation in highest tertile of FVC was no longer significant (OR, 95% CI: 0.8144, 0.4521–1.4672).

**Table 3:** Weighted ORs and 95% CIs for functional limitation according to tertiles of lung function stratified by gender.

	Crude	Model 1	Model 2
**Male**			
**FEV**_**1**_			
<0.76	Ref.	Ref.	Ref.
0.76 to 0.93	0.7159 (0.4623–1.1086)	0.7459 (0.4783–1.1634)	0.7030 (0.4224–1.170)
>0.93	0.4581 (0.3030–0.6928)**	0.5287 (0.3407–0.8204)**	0.5856 (0.3457–0.9920)*
P-trend	<0.001	0.004	0.051
**FVC**			
<1.04	Ref.	Ref.	Ref.
1.04 to 1.26	0.7127 (0.4543–1.1181)	0.7233 (0.4585–1.1411)	0.7022 (0.4152–1.1874)
>1.26	0.3444 (0.2211–0.5365)**	0.3710 (0.2339–0.5885)**	0.3583 (0.2055–0.6247)**
P-trend	<0.001	<0.001	<0.001
**Female**			
**FEV**_**1**_			
<0.76	Ref.	Ref.	Ref.
0.76 to 0.93	0.5084 (0.3783–0.6833)**	0.5485 (0.4038–0.7452)**	0.5961 (0.4130–0.8606)**
>0.93	0.4075 (0.2699–0.6153)**	0.5008 (0.3222–0.7784)**	0.5432 (0.3149–0.9371)*
P-trend	<0.001	<0.001	0.005
**FVC**			
<1.04	Ref.	Ref.	Ref.
1.04 to 1.26	0.4783 (0.3545–0.6554)**	0.5751 (0.4217–0.7844)**	0.5701 (0.3904–0.8324)**
>1.26	0.4217 (0.2664–0.6674)**	0.6008 (0.3678–0.9811)*	0.8144 (0.4521–1.4672)
P-trend	<0.001	0.001	0.063

*P<0.05

**P<0.01. FEV_1_ represents FEV_1_/height^2^, and FVC represents FVC/height^2^.

Model 1 adjusted for age, BMI, smoking status, and stroke.

Model 2 adjusted for more covariates: age, race, education level, marital status, BMI, smoking status, alcohol drinking status, household income, work activity, recreation activity, hypertension, diabetes mellitus, stroke, arthritis, gout, and cancer.

**Fig 1:**
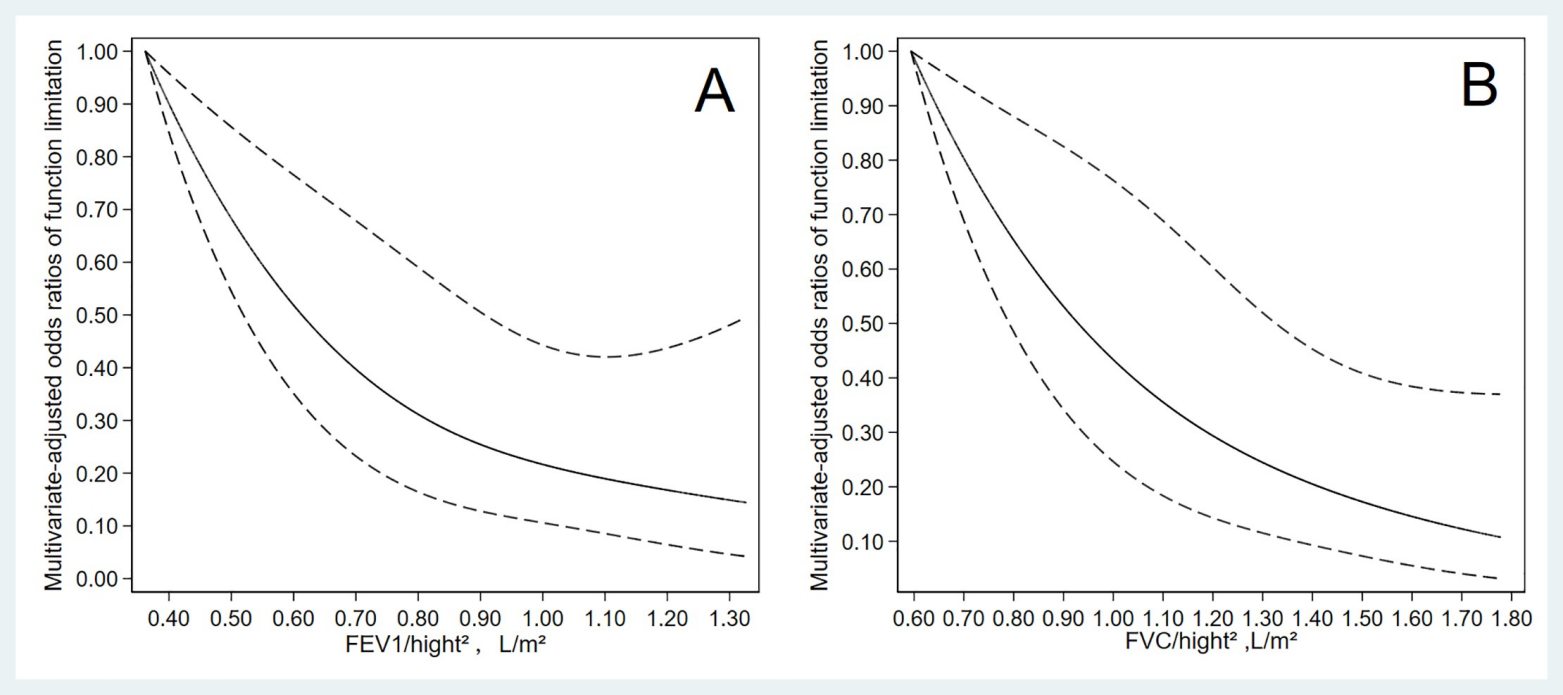
Dose-response relationship between lung function and functional limitation in older adults. The solid line and dash line represent the estimated ORs and their 95% confidence intervals.

Fig 1 displayed the dose-response relationship between lung function and functional limitation among the elderly. In restricted cubic spline models, a nonlinear negative relationship between FEV_1_ and functional limitation was detected (P _for nonlinearity_ <0.001). When the measured value of FEV_1_ increased, the ORs of functional limitation tended to be smaller. The result of restricted cubic spline of FVC and functional limitation was similar to FEV_1_ (P _for nonlinearity_ <0.001).

Taking into account that certain conditions might influence the results of spirometry, we excluded participants with asthma (n = 320), emphysema (n = 52), chronic bronchitis (n = 78), and lung cancer (n = 4) to carry out sensitivity analysis (S1 Table). After removing participants who developed lung diseases and lung cancer, the ORs with 95% CIs of functional limitation based on tertiles of FEV_1_ and FVC remained statistically significant. The inverse associations of FEV_1_ and FVC with functional limitation were coincident.

## Discussion

Based on a representative population in NHANES, our study found that there was a negative association between lung function and functional limitation in older adults, indicating that people with greater lung function had lower odds of being functional limitation. Both stratified analyses and sensitivity analysis confirmed this conclusion. Additionally, the results of restricted cubic spline models examining a non-linear relationship of the decreased ORs of functional limitation with FEV_1_ and FVC increasing further supported our findings. Our results were consistent with those previous studies conducted in patients with pulmonary disease [22, 23], and provided new evidence for the association between lung function and functional limitation in older adults of general population.

Stratified analyses showed that the association of lung function with functional limitation was consistent in different sexes. In males, lung function was inversely associated with functional limitation, whether employing FEV_1_ or FVC. In females, the association between lung function and functional limitation was also negative, although the ORs of functional limitation for the highest tertile of FVC in model 2 did not achieve statistical significance. Meanwhile, we reported a non-linear dose-response relationship between lung function and functional limitation using the restricted cubic spline analysis for the first time. Our findings suggested that the ORs of functional limitation declined as FEV_1_ and FVC increased. Employing sensitivity analysis, excluding participants with some diseases potentially affecting lung function, our results did not alter substantially. The results were agreed in stratified analyses, dose-response analysis, and sensitivity analysis, indicating the stability of this study.

Our results indicated that great lung function may be a protective factor for functional limitation in older adults. Interventions and health promoting strategies on maintaining good lung function are meaningful of older adults to avoiding functional limitation. In clinical practice, most treatments, such as chest physiotherapy, drug therapy, and pulmonary rehabilitation, have been proven to promote the recovery of lung function in patients with acute and chronic lung diseases [30–33]. In addition, breathing training, nutrient intake, physical activity may improve lung function in healthy adults [34–36]. Future studies on the improvement of lung function in older people may help to reduce the odds of being functional limitation.

Although several studies have evaluated the relationship between lung function and functional limitation among the patients with lung function impairment, this association has not previously been assessed, to our knowledge, in a general population of older adults. A retrospective study conducted by Diane et al. showed the protective role of preserved lung function on functional limitation (defined by the 6 minute walk distance test) in patients with dyspnea [22]. A cross-sectional study by Francesc et al. reported that FEV_1_ was negatively associated with self-reported mobility limitation in patients with COPD [23]. Furthermore, a number of studies have been conducted on the relationship of lung function with disability. A cross-sectional study by Thorpe et al. suggested that impaired lung function in terms of percent predicted peak expiratory flow (PEF), was more likely with disability in women [37]. A cohort study by Aron et al. noted that pulmonary function was related to mobility disability defined by gait speed [38]. Most of previous studies conducted in patients with lung function impairment supported our findings.

There are some possible explanations and underlying mechanisms for this association. Firstly, lung function and metabolism are closely related. It has been reported that lung function impairment was related to insulin resistance, the metabolic syndrome, and obesity [39–41]. Changes in weight and metabolism and their associated diabetes, cardiovascular disease, and stroke may be important reasons for difficulties with mobility [42, 43]. Secondly, muscle dysfunction is a common manifestation of COPD [44] with reduction in muscle mass and muscle fiber degeneration, related to chronic hypoxia and metabolic derangements, the main reasons for difficulty in maintaining activity [45–47]. Thirdly, inflammatory factors such as, C-reactive protein, tumor necrosis factor alpha (TNF-α) and interleukin-6 (IL-6) may mediate the link between lung function with functional limitation [48–51]. Catabolic effects of proinflammatory markers on the muscle may contribute to functional decline [52].

Major strengths of this study are assessment of the association and the dose-response relationship between lung function and functional limitation in older adults at the first time. Furthermore, the results of stratified analysis by gender and sensitivity analysis are consistent. In addition, we have adjusted for many covariates to yield more credible and reliable results. However, some limitations in our study merit mention. Firstly, because of the cross-sectional design, the causality between lung function and functional limitation was not identified. At present, due to no evidence in reverse association, the possibility that functional limitation or disability leads to impaired lung function remains uncertain. Future prospective longitudinal studies are needed to confirm causality. Secondly, although we have adjusted potential confounders as far as possible, other relevant factors, such as nutrition, have not been taken into account. Thirdly, the percent predicted value was a common indicator of lung function in clinical, whereas the data was not available in NHANES. Then FEV_1_ and FVC adjusted height-squared were analyzed instead. Fourthly, we excluded diseases affecting lung function as far as possible in the sensitivity analysis, but some diseases unevaluated in NHANES, such as interstitial lung diseases, were not eliminated.

## Conclusions

In conclusion, our study indicated an inverse association with an apparently non-linear dose-response relationship between lung function and functional limitation in older adults. Further prospective studies are required to confirm our findings and causality of the relationship in the future.

## Supporting information

S1 Table Weighted ORs and 95% CIs for function limitation excluding lung diseases and lung cancer, NHANES 2007–2012. (DOC)
