# Supplementary material for: Association of lung function with functional limitation in older adults: A cross-sectional study
Source: PLoS One. 2021 Jun 29;16(6):e0253606. doi: 10.1371/journal.pone.0253606 (PMC8241026; doi:10.1371/journal.pone.0253606)
Supplement: S1 Table — (DOC) [file pone.0253606.s001.doc]

| **S1** **Table Weighted ORs and 95% CIs for function limitation excluding lung diseases and lung cancer, NHANES 2007–2012** | | | |
| --- | --- | --- | --- |
|  | Crude | Model 1 | Model 2 |
| **FEV1** |  |  |  |
| ＜0.76 | Ref. | Ref. | Ref. |
| 0.76 to 0.93 | 0.5427 (0.3581-0.8226)** | 0.6287 (0.4016-0.9842)* | 0.6039 (0.3822-0.9544)* |
| ＞0.93 | 0.3281 (0.2299-0.4681)** | 0.5173 (0.3105-0.8619)* | 0.5397 (0.3053-0.9541)* |
| P-trend | ＜0.001 | 0.011 | 0.027 |
| **FVC** |  |  |  |
| ＜1.04 | Ref. | Ref. | Ref. |
| 1.04 to 1.26 | 0.5048 (0.3630-0.7020)** | 0.6204 (0.4550-0.8460)** | 0.6120 (0.4115-0.9101)* |
| ＞1.26 | 0.2667 (0.1829-0.3889)** | 0.4245 (0.2784-0.6472)* | 0.5345 (0.3065-0.9320)* |
| P-trend | ＜0.001 | ＜0.001 | 0.022 |
| *P＜0.05, **P＜0.01. FEV1 represents FEV1/height², and FVC represents FVC/height².  Model 1 was adjusted for age, gender, BMI, smoking status and stroke.  Model 2 was adjusted for more covariates: age, gender, race, education level, marital status, BMI, smoking status, alcohol drinking status, household income, work activity, recreation activity, hypertension, diabetes mellitus, stroke, arthritis and gout. | | | |
